# Strawberry Additive Increases Nicotine Vapor Sampling and Systemic Exposure But Does Not Enhance Pavlovian-Based Nicotine Reward in Mice

**DOI:** 10.1523/ENEURO.0390-22.2023

**Published:** 2023-06-12

**Authors:** Theresa Patten, Natalie L. Johnson, Jessica K. Shaw, Amanda M. Dossat, Allison Dreier, Bruce A. Kimball, Daniel W. Wesson, Mariella De Biasi

**Affiliations:** 1 Department of Psychiatry, University of Pennsylvania, Philadelphia, Pennsylvania 19104; 2 Department of Neuroscience, University of Pennsylvania, Philadelphia, Pennsylvania 19104; 3 Pharmacology Graduate Group, University of Pennsylvania, Philadelphia, Pennsylvania 19104; 4 School of Arts and Sciences, Perelman School of Medicine, University of Pennsylvania, Philadelphia, Pennsylvania 19104; 5 Department of Pharmacology and Therapeutics, Center for Smell and Taste, Center for Addiction Research and Education, University of Florida College of Medicine, Gainesville, Florida 32610; 6 Monell Chemical Senses Center, Philadelphia, Pennsylvania 19104

**Keywords:** chemosensory, e-cigarettes, flavor additives, nicotine exposure, nicotine reward

## Abstract

Nicotine is an addictive drug whose popularity has recently increased, particularly among adolescents, because of the availability of electronic nicotine devices (i.e., “vaping”) and nicotine e-liquids containing additives with rich chemosensory properties. Some efforts to understand the role of these additives in nicotine reward suggest that they increase nicotine reward and reinforcement, but the sensory contributions of additives, especially in their vapor forms, are largely untested. Here, to better understand how a fruit-flavored (i.e., strawberry) additive influences nicotine reward and aversion, we used a conditioned place preference (CPP) procedure in which nicotine and a strawberry additive were delivered as a vapor to male and female adolescent mice. We found that nicotine vapor alone can lead to a dose-dependent CPP when using a biased design. The strawberry additive did not produce CPP on its own, and we did not observe an effect of the strawberry additive on nicotine vapor-induced reward. Nevertheless, mice exposed to nicotine plus strawberry additive vapor had higher plasma cotinine concentrations, which did not appear to reflect altered nicotine metabolism. Instead, by directly measuring vapor sampling through respiration monitoring, we uncovered an increase in the amount of sniffing toward strawberry-containing nicotine vapor compared with nicotine vapor alone. Together these data indicate that chemosensory-rich e-liquid additives may enhance the perceived sensory profile of nicotine vapors rather than the reward value per se, which leads to overall increased nicotine exposure.

## Significance Statement

With the rise in popularity of flavored e-cigarette products, many have considered the possibility that flavor volatiles will enhance nicotine reward; however, the possibility that flavor additives have chemosensory properties that can affect nicotine intake has been largely overlooked. Here, by delivering nicotine to adolescent mice as a vapor, we were able to consider both possibilities. We found that mice had increased sniffing intensity and nicotine exposure when vapors contained a strawberry additive despite the fact that the same additive was unable to enhance pavlovian nicotine reward using a conditioned place preference paradigm. This research highlights the importance of considering the chemosensory properties of e-cigarette additives as a mechanism for their effect on nicotine use.

## Introduction

The number of adolescent nicotine users in the United States increased in 2019 for the first time in decades, an effect driven by a rise in e-cigarette use (see Centers for Disease Control and Prevention report at: https://www.cdc.gov/media/releases/2019/p0211-youth-tobacco-use-increased.html). Chemosensory-rich additives in e-cigarette liquids, also known as “flavors,” promote nicotine use in humans, possibly because of reduced perceptions of harm (Ford et al., 2016; Pepper et al., 2016; Chaffee et al., 2018). In addition, sweet additives such as those engineered to be perceived as fruit like or candy like are attractive to adolescents and young adults who have a higher preference for sweetness and sensory cues, including odors, associated with sweetness (Desor and Beauchamp, 1987; Zandstra and de Graaf, 1998; Hoffman et al., 2016).

Chronic nicotine exposure via combustible cigarettes is associated with serious health conditions and is the leading cause of preventable death in the developed world (Creamer et al., 2019). Given that e-cigarette use is associated with a progression to combustible cigarette use, there is concern that e-cigarettes and their attractive additives have ushered in another generation of individuals who will come to struggle with physical and psychological health complications, such as an increased likelihood of drug dependence and an increased risk of cognitive deficits (Goriounova and Mansvelder, 2012; Soneji et al., 2017).

E-cigarette additives with significant chemosensory properties are widely hypothesized to increase nicotine reward (Audrain-McGovern et al., 2016; Goldenson et al., 2016; Kim et al., 2016; Leventhal et al., 2019b; Patten and De Biasi, 2020), as sensory perception plays a role in nicotine reward, use, and craving. Blocking the airway sensory experience of smoking with a local anesthetic reduces smoking satisfaction and sensory stimulation with other irritants (e.g., citric acid) can reduce cigarette cravings (Rose et al., 1984; Levin et al., 1990). E-cigarettes with fruity chemical additives are perceived as sweet, and flavor-enhanced sweetness and “liking” are often correlated in clinical reports of subjective reward (Goldenson et al., 2016; Kim et al., 2016; Leventhal et al., 2019b). “Sweetness” is rewarding and reinforcing and can support both conditioned place preference (CPP) and self-administration in rodents (Bacon et al., 1962; Dufour and Arnold, 1966). It is possible that the sweet reward contributed by e-cigarette additives synergizes with the nicotine reward, enhancing overall reward. Users also report that flavored additives mask the harshness of the cigarette taste and that the addition of a fruit additive to nicotine-containing e-cigarettes suppresses unappealing sensations (Kim et al., 2016; Chen et al., 2019; Leventhal et al., 2019a).

We investigated the role of a strawberry flavored e-cigarette liquid on nicotine reward using a CPP paradigm in which nicotine was delivered to adolescent mice as a vapor. This route of drug delivery during Pavlovian conditioning allowed us to simultaneously consider the pharmacological and sensory components of e-cigarette reward and aversion. We next monitored the inhalation patterns of mice as they engaged with nicotine vapor with or without a strawberry additive. Together, our results begin to unveil novel sensory influences on e-cigarette use, preference, and reinforcement.

## Materials and Methods

### Animals

**Figure 1.**
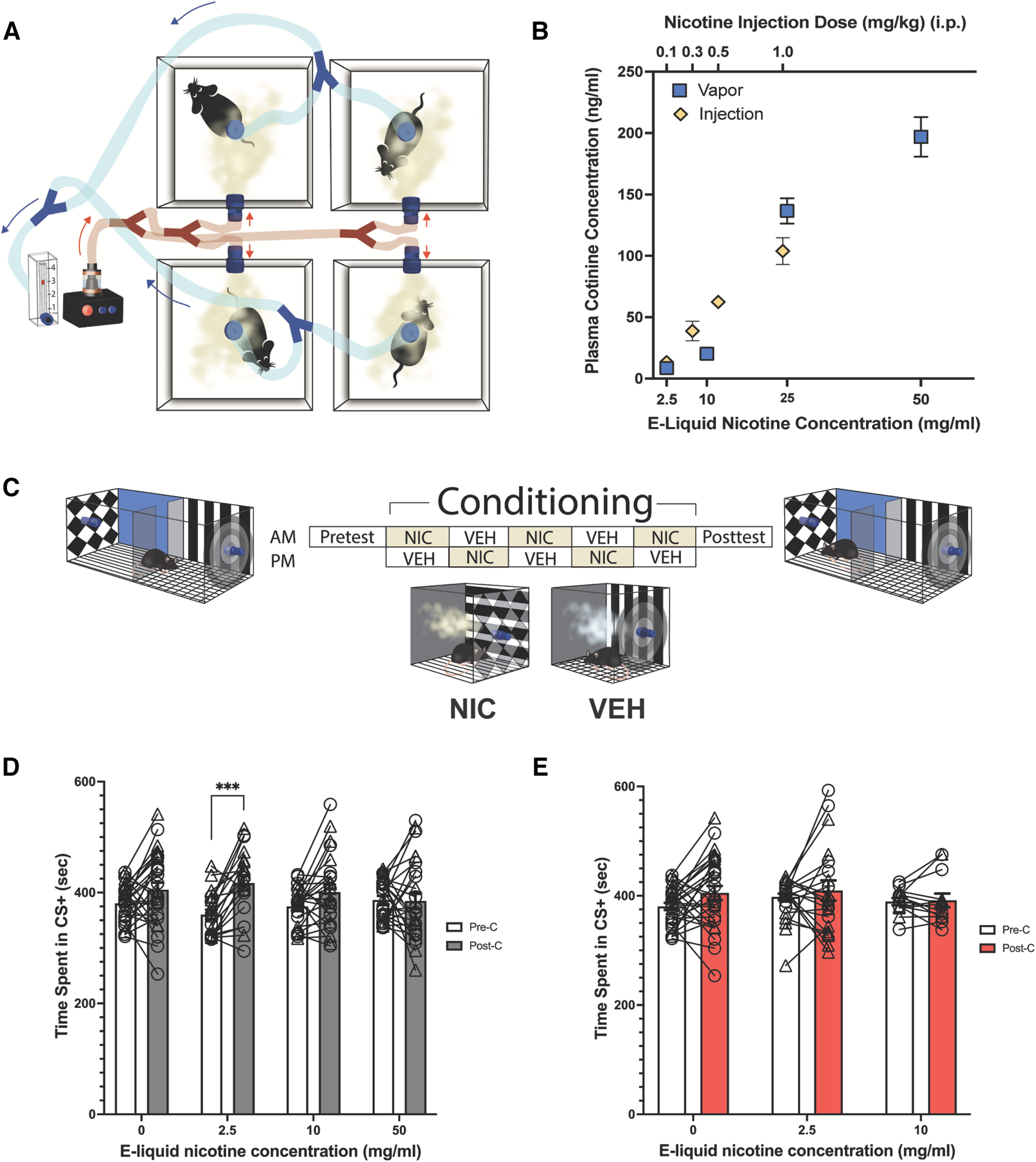
Nicotine delivery via e-cigarette vapor results in measurable plasma cotinine concentrations, and the rewarding properties of e-cigarette vapors can be evaluated using conditioned place preference. ***A***, A vapor exposure setup allows for the simultaneous delivery of e-cigarette vapor to four mice, each in its own airtight chamber. ***B***, Scatter plots of plasma cotinine concentrations detectable in adolescent mice (mean ± SEM) 30 min after nicotine exposure via an intraperitoneal injection (yellow diamonds, top *x*-axis, *n* = 7, 4, 7, 5) or e-cigarette vapor exposure as depicted in ***A*** (blue boxes, bottom *x*-axis, *n* = 27, 28, 4, 46). ***C***, Schematic of the vapor-conditioned place preference paradigm in which mice receive experimental vapor (nicotine) once per day for 5 d in the CS+ compartment and vehicle once per day for 5 d in the CS– compartment. ***D***, Before and after plots show the time spent in the CS+ environment before and after conditioning with nicotine vapor (*n* = 28, 20, 24, 25). ***E***, Before and after plots show the time spent in the CS+ environment before and after conditioning with strawberry vapor of various concentrations (*n* = 28, 20, 13). In ***D*** and ***E***, bars show the mean ± SEM, and the vehicle data (i.e., “0”) are the same in both panels. Two-way repeated-measures ANOVA and multiple comparisons: ***p < 0.001, relative to time spent in CS+ before conditioning. For all charts, circles represent individual male mice and triangles represent female mice. NIC, Nicotine; VEH, vehicle.

**Figure 2.**
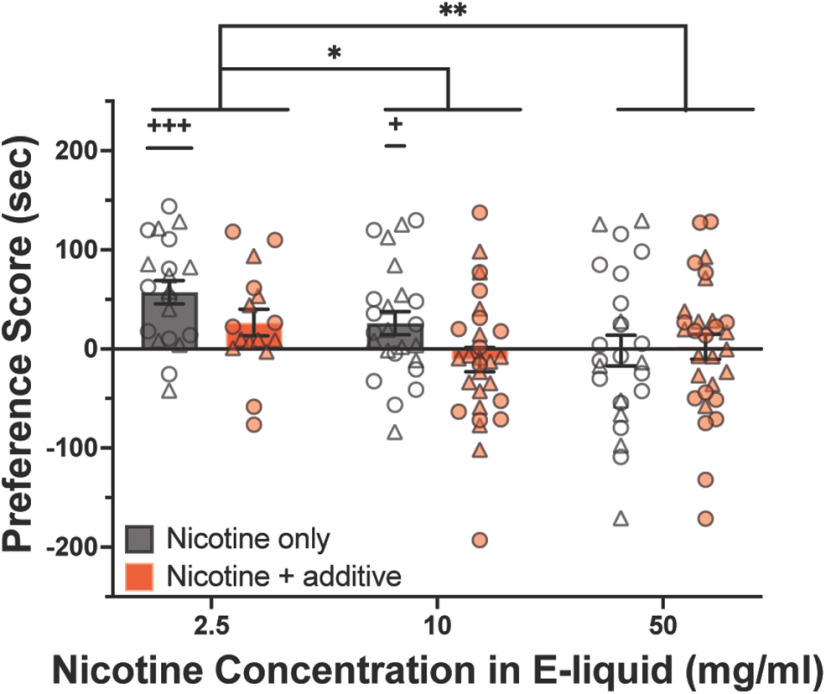
Strawberry additive does not affect nicotine CPP. Adolescent mice were conditioned to nicotine vapor with (2.5% strawberry flavorant) or without a strawberry additive using a CPP-biased protocol (nicotine only, *n* = 20, 24, 25; nicotine + additive, *n* = 16, 29, 30). Scatter plots represent individual mice, and bars represent the mean ± SEM. One-sample *t* tests (vs 0 s change in time spent in CS+): +*p* < 0.05, +++*p* < 0.001. Two-way ANOVA and multiple comparisons: *p < 0.05, **p < 0.01. For all charts, circles represent individual male mice and triangles represent female mice.

**Figure 3.**
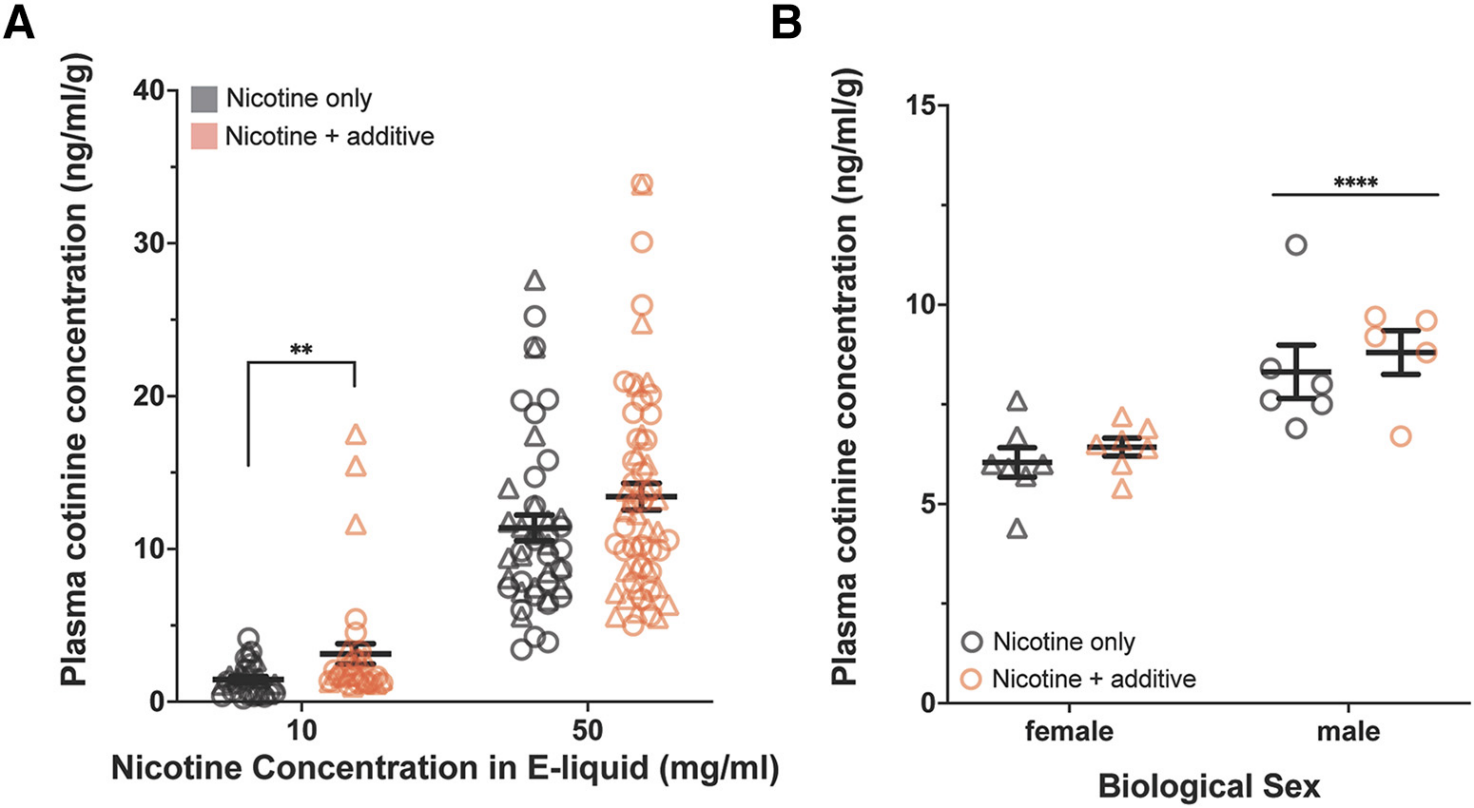
Increased plasma cotinine levels in mice exposed to strawberry nicotine vapors, a proxy for increased nicotine exposure. ***A***, Mice exposed to vapor containing a strawberry-additive and nicotine e-liquid (10 or 50 mg/ml) had higher plasma cotinine concentrations relative to their body weight (in ng/ml/g) than mice exposed to nicotine-only vapor (10 mg/ml nicotine: *n* = 28; +additive, 34; 50 mg/ml nicotine: *n* = 46; +additive, 60). ***B***, Adolescent mice were interperitoneally injected with 1.0 mg/kg nicotine in PBS with or without 2.5% strawberry additive. The presence of strawberry additive in the injection vehicle did not influence plasma cotinine concentrations 30 min postinjection; however, there was a significant effect of sex where, overall, male mice had higher plasma cotinine concentrations 30 min post-nicotine injection (females: *n* = 7, 7; males: *n* = 6, 5). **p < 0.01, ****p < 0.0001. Circles represent individual male mice, and triangles represent female mice.

**Figure 4.**
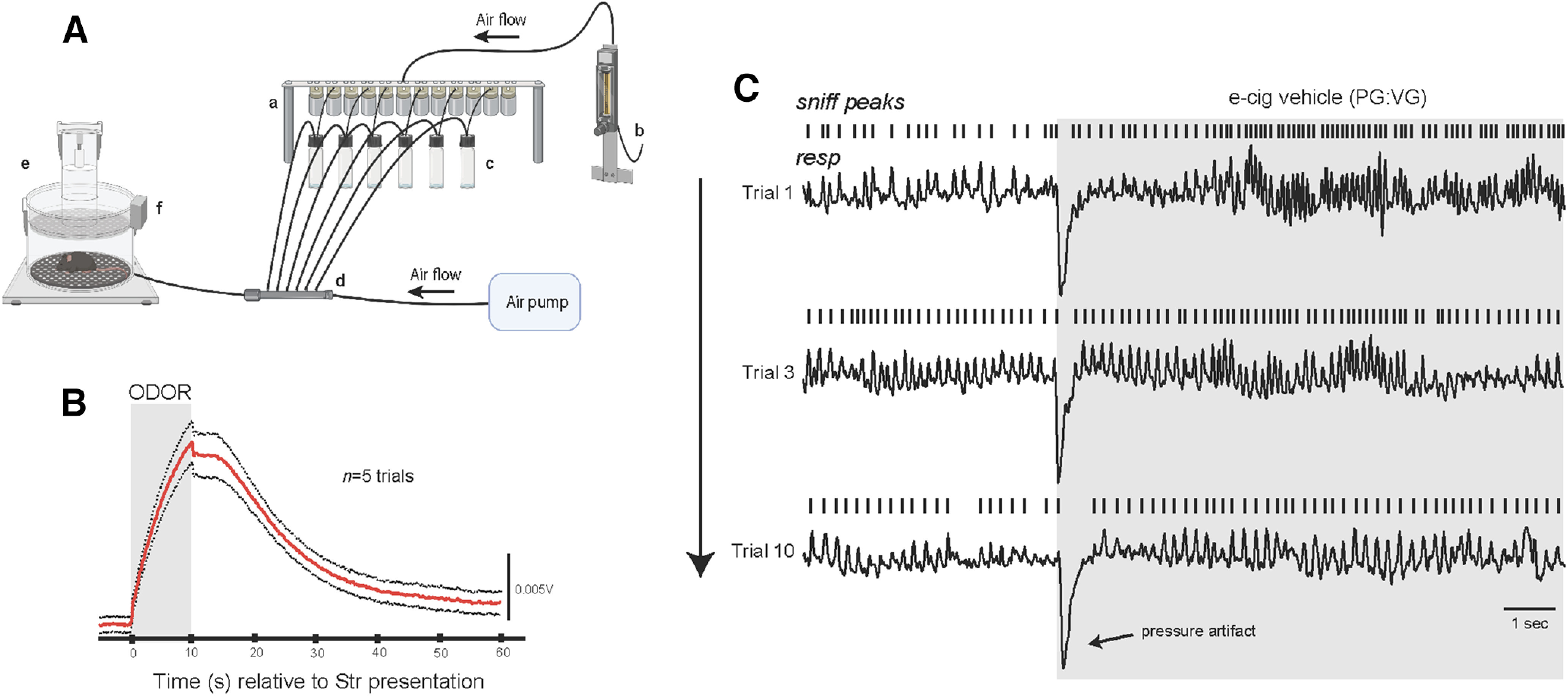
Paradigm for monitoring the sniffing of mice to e-cigarette odors. ***A***, Design of the system. Openings of computer-controlled valves (a) allows for the flow of air as regulated by a flowmeter (b) to pass through its respective valve into the connected odor vial (c). Airflow continues for 10 s, flowing from the headspace of the vial containing liquid odor through the connected tubing to ultimately combine with constant airflow in a manifold (d) and then to the mouse in the plethysmograph (e). Odor enters from the base of the plethysmograph, beneath the perforated floor. Changes in air pressure within the plethysmograph (indicating respiration) are detected by a flow transducer and digitized (f). Image was made with BioRender. ***B***, The average of 5 trails of 10 s presentations of 2.5% strawberry (Str) e-liquid odor acquired via a photoionization detector (Aurora Scientific) illustrating onset and evacuation of odor from within the plethysmograph. Timing of strawberry odor delivery is indicated by gray box. ***C***, Representative respiratory traces from a single mouse throughout presentations of PG/VG. Rasters indicate sniff peaks. Timing of PG/VG delivery is indicated by gray box.

**Figure 5.**
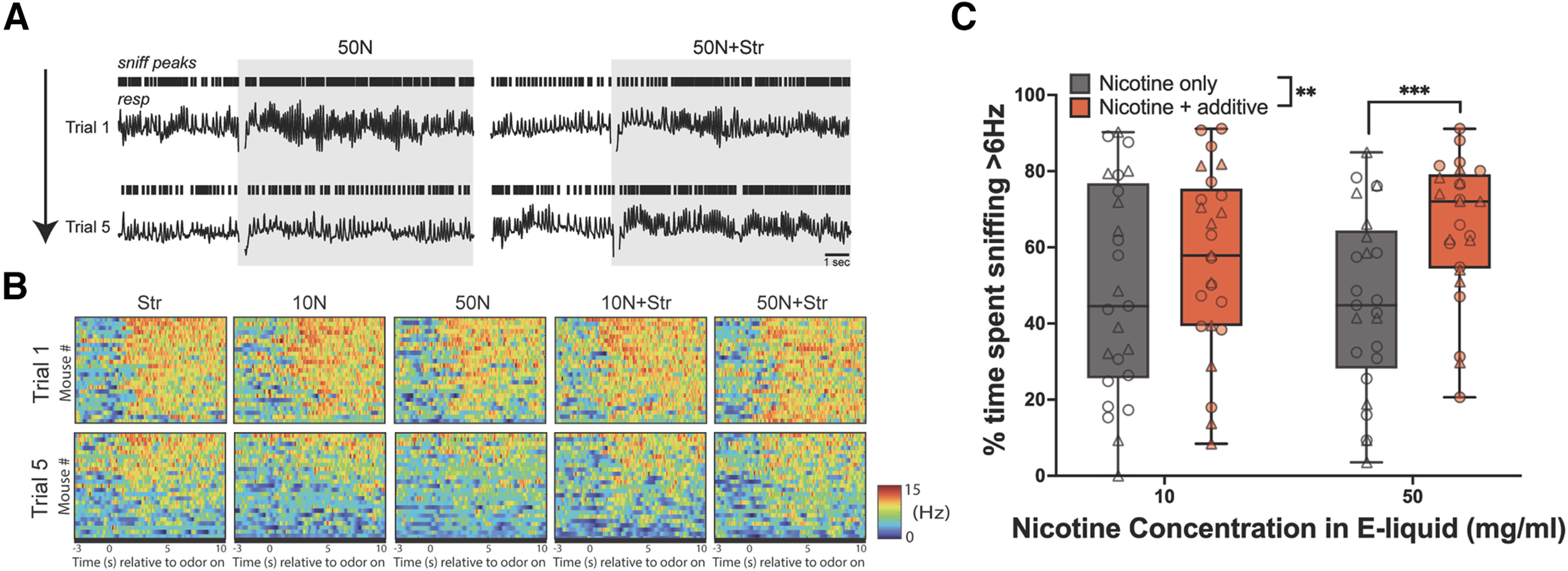
The addition of strawberry e-cigarette additive promotes the inhalation of nicotine vapor. ***A***, Example respiratory traces from one mouse showing its sniffing in response to pseudorandom presentations of 50 mg/ml nicotine (50N; left) and 50 mg/ml nicotine with the strawberry additive (50N+Str; right). Rasters indicate sniff peaks. ***B***, Sniffing dynamics of all mice during the first and fifth presentation of all e-cigarette odors. 2-D histograms depict the average sniffing frequency across the 10 s of odor presentation. Data are organized in descending order based on average sniff frequency during odor presentation. Dashed line indicates odor onset. ***C***, Percentage of time spent sniffing by mice during presentation with flavored and unflavored nicotine vapors. Two-way ANOVA detected a main effect of flavor, and multiple-comparisons test determined that mice spent significantly more time in investigatory sniffing when presented with 50N+Str. *N* = 25, **p < 0.01 and ***p < 0.001 for all comparisons. In ***C***, circles represent individual male mice and triangles represent female mice.

Adolescent male (*n* = 191) and female (*n* = 170) C57BL/6J mice between postnatal day 28 (P28) and P49, corresponding approximately to humans 12–18 years of age (Yuan et al., 2015), were used in the CPP and blood collection experiments (Figs. 1-5). They were housed at the University of Pennsylvania with a reverse 12 h light/dark cycle (lights off at 10:00 A.M.) in a temperature-controlled room (24 ± 2°C; relative humidity, 55 ± 10%). All behavioral testing and e-cigarette vapor exposures occurred in the dark phase of the light cycle. Animals were either purchased from The Jackson Laboratory or bred in-house. Those bred in-house were the offspring of 20+ different breeding pairs and distributed throughout treatment groups to ensure adequate genetic diversity. Similarly, adolescent male (*n* = 14) and female (*n* = 11) C57BL/6J mice between P28 and P49 were used in the plethysmography experiments (Figs. 4, 5) and were housed at the University of Florida in comparable conditions. All mice in this study had *ad libitum* access to food and water. All procedures were approved by the appropriate Institutional Animal Care and Use Committee and followed the guidelines for animal intramural research from the National Institutes of Health.

10.1523/ENEURO.0390-22.2023.f1-1 Figure 1-1 GC/MS detects chemical volatiles in strawberry e-liquid. The chromatogram from GC/MS analysis of 0.1% “Strawberry Flavor Concentrate” (Liquid Barn) in water shows the following chemicals dominate in the commercial e-liquid headspace in order from highest to lowest chromatographic peak responses: (a) ethyl butyrate, (b) 2-methyl-ethyl butyrate, (c) benzyl acetate, (d) propyl butyrate, (e) 3-hexen-1-ol, (f) 3-hexenyl acetate, (g) linalool, (h) menthyl acetate, and (i) benzyl butyrate. Download Figure 1-1, TIF file.

10.1523/ENEURO.0390-22.2023.f1-2 Figure 1-2 pH of the e-liquids utilized in this study estimated by assuming [H^+^] in aqueous solution. Briefly, e-liquids were made in a 50:50 VG/PG blend with nicotine dissolved in the VG portion and additive dissolved in the PG portion. To reduce the viscosity of the e-liquid and in order to mimic the protocol used in the study by St. Helen et al. (2017), a 1:10 e-liquid-to-Milli-Q water dilution of each e-liquid was prepared. pH test strips were then used to estimate the pH. Download Figure 1-2, DOC file.

### E-liquid and nicotine preparations

E-liquid materials [Vegetable Glycerin (VG), Propylene Glycol (PG), NicSelect Nicotine (free-base; 100 mg/ml in VG), and Strawberry Flavor Concentrate (in PG)] were purchased from Liquid Barn. E-liquids were mixed in the laboratory to the desired concentration of nicotine and strawberry additive while maintaining a 50:50 ratio of VG/PG. The e-liquid was kept in the dark and made fresh every 3 d to prevent nicotine degradation. pH was not adjusted between solutions, but adding the flavor additive to the nicotine solution slightly lowered the pH of the e-liquid (Figs. 1, 2). For intraperitoneal nicotine injections, nicotine tartrate salt (Sigma-Aldrich) was dissolved in PBS to desired concentrations (0.03–0.1 mg/ml freebase nicotine). When the additive was added to PBS for injections, a concentrated nicotine stock solution was used to make both the nicotine only and nicotine plus additive injection solutions. The final injection solution was diluted with either PBS alone or PBS containing 5% strawberry additive (Liquid Barn) to ensure that nicotine concentration in the injection solutions was identical.

### E-cigarette vapor delivery

E-liquid was vaporized using a Vapor Generator Controller (La Jolla Research) in conjunction with a SMOK Baby Beast Brother e-cigarette tank and V8 X-Baby-Q2 coils (0.4 Ω; SMOK). The Vapor Generator Controller was operated at 75.0 W using the preset “nicotine” settings in the system, which has the temperature set to 400°F. This machine has been previously validated for nicotine vapor delivery (Montanari et al., 2020; Cooper et al., 2021). For all vapor exposures (i.e., for cotinine quantification and for the conditioning phase of CPP experiments), the vapor “puff” from a single e-cigarette is diverted into four air-tight exposure apparatuses (dimensions = 5.25 × 5.25 × 5.625 inches) and is quickly replaced by clean room air via a vacuum (flow rate = 3.0 L/min; Fig. 1*A*, schematic). We delivered a 1 s puff every 90 s for a total of 25 min, resulting in a vapor period, which, in pilot experiments, we found was sufficient to allow the delivery of pharmacologically relevant doses of nicotine while delivering vapor in a puff-like pattern. This delivery pattern also provided sufficient time for the perception of the cues and the association of cues with the presence (or absence) of nicotine. The 25 min vapor delivery period is followed by a 5 min “washout” to protect experimenters from vapor inhalation. Therefore, mice are placed individually into one of the four exposure chambers for a total of 30 min per exposure. When vapor exposures were part of a conditioned place preference or aversion experiment, tactile and visual cues were added to the floor and to the outside of the clear Plexiglas chamber (Fig. 1*C*). Tactile cues comprised either rigid embroidery mesh in a grid pattern or flexible corrugated floors.

### Cotinine quantification

To measure plasma cotinine (the primary metabolite of nicotine) concentrations in mice following nicotine exposures, trunk blood was collected 30 min following nicotine treatment. Thirty minutes postinjection has been previously shown to be the approximate time at which the maximum concentration of plasma cotinine is achieved in C57BL/6 mice (Siu and Tyndale, 2007), and pilot studies in our laboratory indicate that this is also true using our vapor delivery system (T. Patten, A. Dreier, M. De Biasi, unpublished observations). After blood collection and plasma separation, cotinine was quantified using a Mouse/Rat Cotinine ELISA kit according to package instructions (CALBIOTECH). Samples were diluted with purified, Milli-Q water when necessary to achieve a concentration of cotinine within the range of the standard curve that allowed for the most accurate cotinine quantification (10–50 ng/ml), and a pipetting variability of <15% was required for the inclusion of data. Samples that did not meet these requirements were reanalyzed. Cotinine values in diluted samples were calculated as follows: cotinine = cotinine_dilution_ * dilution factor.

### Analyses of strawberry additive volatiles and e-liquid nicotine concentration

To identify major volatiles in the Strawberry Flavor Concentrate (Liquid Barn), headspace gas chromatography/mass spectrometry (GC/MS) analyses were conducted with a dynamic headspace analyzer (HT3 Automated Headspace Analyzer, Teledyne Tekmar) outfitted with a thermal desorption trap (Supelco Trap K Vocarb 3000 Thermal Desorption Trap, Sigma-Aldrich). The GC/MS (Trace Ultra, Thermo Fisher Scientific) was equipped with a single quadrupole mass spectrometer (Thermo Fisher Scientific) and a 30 m × 0.25 mm ID fused-silica capillary column (Stabiliwax-DA, Restek). The e-liquid additive solutions (0.1% in water) were subjected to dynamic headspace GC/MS analysis by placing 50 μl in a sealed 20 ml headspace vial. The vial was maintained at 30°C, swept with helium for 10 min (flow rate, 75 ml/min), and the volatiles were collected on the thermal desorption trap. Trap contents were desorbed at 260°C directly into the GC/MS using a split injection. The GC oven program had an initial temperature of 40°C (held for 3.0 min) followed by a ramp of 7.0°C/min to a final temperature of 230°C (held for 6.0 min). The MS was used in scan mode with a mass/charge ratio from 33 to 400 using a 3 min solvent delay. Mass spectral peak identifications were assigned based on the library search of the National Institute of Standards and Technology Standard Reference Database.

### Nicotine vapor-conditioned place preference

The CPP procedure is divided into the following three phases: pretest, conditioning, and post-test (Fig. 1*C*). All phases of testing occurred during the dark cycle in either dim light (1–2 lux) or red light to prevent circadian rhythm disruption. During the pretest, mice were able to freely explore a testing chamber with two rooms containing identical cues (e.g., tactile, visual) and dimensions as the vapor exposure chamber (described in subsection E-cigarette vapor delivery) for 15 min. One chamber had the following cues: walls, black and white “bulls-eye” symbol; solid white, vertical black and white stripes; and solid black; and flooring: rigid white embroidery mesh in a grid pattern. The opposing chamber had the following cues: walls. black and white argyle pattern; solid blue, horizontal black and white stripes; and solid black; and flooring: flexible corrugated floors. CPP was conducted using a biased design (i.e., nicotine was assigned to the least preferred chamber). During the 5 d conditioning phase, mice received one CS+ (conditioned rewarded stimulus) and one CS– (conditioned unrewarded stimulus) conditioning session each day, in which they were placed in an exposure chamber individually with appropriate cues and received either control (50/50 VG/PG blend; vehicle) vapor, strawberry vapor, or nicotine with or without strawberry-flavored vapor. A control group received vehicle vapor in both compartments. A.M. and P.M. exposure sessions occurred ∼4 h apart. Following conditioning, mice were placed back into the testing chamber and allowed to freely explore both chambers for 15 min in a drug-free state. To avoid extreme bias skewing the results, mice showing strong innate preference (i.e., >65%) toward either compartment during the initial pretest were excluded.

Videos of both the pretest and post-test were recorded, and an experimenter blinded to the test stage (pretest or post-test) and the treatment group scored the time spent in each compartment, as well as the time spent climbing in the center region (unfocused). Preference scores were determined by comparing the time spent in the treatment-paired compartment (CS+) before and after conditioning with e-cigarette vapors (i.e., preference score = time in CS+ after conditioning – time in CS+ before conditioning), as this allows for a detection in the change in preference (for review, see McKendrick and Graziane, 2020).

### Cotinine category determination

As the inclusion of the strawberry vapor increased nicotine intake (measured by cotinine plasma levels), thereby altering the dose of nicotine received, we sought to compare mice based on the actual level of nicotine exposure. “Cotinine categories” were objectively defined according to plasma cotinine concentrations following the injection of nicotine doses previously shown to be “subthreshold” (0.1 mg/kg), “rewarding” (0.3–0.5 mg/kg), and “aversive” (1.0 mg/kg) in rodents (Fudala et al., 1985; Jorenby et al., 1990; Torres et al., 2008). In our injection studies, mean ± SD plasma cotinine concentrations following a 0.3 mg/kg injection were 47.75 ± 19.05 ng/ml. We therefore set the lower threshold of the cotinine concentration defined as rewarding at 28.7 ng/ml (i.e., 1 SD below the average cotinine concentration of a rewarding 0.3 mg/kg nicotine injection). The upper threshold of rewarding plasma cotinine concentrations was set to 91.35 ng/ml, 1 SD above the average cotinine concentration following a rewarding 0.5 mg/kg, intraperitoneal (i.p.) nicotine injection, thereby ensuring that we captured a large range of cotinine values associated with a rewarding dose of nicotine. Subthreshold cotinine concentrations were determined to be anything below the rewarding threshold (i.e., <28.7 ng/ml), and aversive cotinine concentrations were defined as cotinine concentrations above the rewarding threshold (<91.35 ng/ml).

### Plethysmography

E-liquids were prepared as above, and their volatile odors were presented to the mice through an air dilution olfactometer (Fig. 4*A*). During experimentation, all stimuli were contained in 40 ml glass headspace vials at room temperature. Odors were mixed with medical-grade nitrogen (Airgas) passed through headspace vials at a flow rate of 50 ml/min, following which they were mixed with a carrier stream of clean, filtered room air (1 L/min; Tetra Whisper air filter) before being introduced into the plethysmograph. All odors were handled within independent tubing to prevent cross-contamination.

To monitor the respiration of mice in response to e-cigarette odors, we used whole-body plethysmography and a computer-controlled olfactometer (Johnson et al., 2020). The unrestrained whole-body plethysmograph (Data Sciences International) allowed for the detection of respiratory transients as mice freely explored the chamber. Transients were detected using a flow transducer (Data Sciences International) and digitized at 300 Hz (bandpass filter, 0.1–12 Hz; Synapse software, Tucker Davis Technologies), following a 500× gain amplification (CYGNUS Technologies). The olfactometer allowed for precise control of odor delivery into the plethysmograph chamber (Fig. 4*B*). User-initiated openings of valves (Parker Hannifin) via a computer and digital relay (LabJack) allowed for the flow of vapors into the bottom of the plethysmograph for perception by the mouse. Following each stimulus trial, odor-vaporized air was passively cleared from the plethysmograph through an exhaust outlet at the ceiling of the chamber since the filtered room air is continuously delivered.

Mice were acclimated to the plethysmograph for 2 d before testing. During acclimation, mice were placed in the plethysmograph for 30 min and vehicle solution (50/50 VG/PG) was presented for 10 s each with a 60 s intertrial interval (ITI). The goal of this was to allow the mice to acclimate to handling, the chamber, and other possible nonolfactory cues (e.g., pressure changes, sounds) associated with valve opening and airflow. The day following this acclimation, mice were exposed to e-cigarette odors. On this day, first VG/PG was presented for 10 s with a 60 s ITI to further acclimate the mouse to vapor delivery, followed by five trials of pseudorandom presentations of 2.5% strawberry, 10 mg/ml nicotine ± 2.5% strawberry, and 50 mg/ml ± 2.5% strawberry. Each odor was presented for 10 s with a 90 s ITI.

Inhalation peaks were detected offline in Spike2 (Cambridge Electronic Design). First, the respiratory data were filtered (bandpass filter, 0.1–12 Hz). Second, the maximum point of each respiratory cycle was identified, and instantaneous frequency was then calculated based off of the duration between one peak and its preceding peak. Data were downsampled to 50 Hz for subsequent analyses. Although the odor valves were open for 10 s, the first second was excluded from analysis to account for the latency of odor delivery to the plethysmograph (Fig. 5*B*) and unavoidable pressure artifacts (Fig. 5*C*). We calculated the average instantaneous frequency over the remaining 9 s of each odor presentation to quantify odor-evoked sniffing. Amplitude was calculated based on the root mean square of the respiratory signal.

### Statistical analyses

All datasets were tested for normality using the D’Agostino–Pearson test before selecting appropriate methods of analysis (nonparametric vs parametric). Nonparametric data (i.e., data that were not normally distributed) were analyzed using Mann–Whitney and Wilcoxon tests, and the Scheirer–Ray–Hare test—an extension of the Kruskal–Wallis test that allows for a factorial design—was used to detect interactions. The parametric tests used in this study include *t* tests and fixed-effects one and two-way Analysis of Variance (ANOVAs). Summary data is represented in figures as the mean +/− the standard error of the mean (SEM). GraphPad Prism was used for all data analyses except the Scheirer–Ray–Hare test, which was conducted using the Real Statistics Add-In for Microsoft Excel. In all figures, asterisks (*) indicate a significant difference from a comparison group, a plus sign (+) indicates a significant difference from 0 (0 s change from pretest; no change in preference from baseline). Mice were excluded from CPP experiments in one of two instances: 20% of screened mice (*n* = 39 of 194) showed a strong initial preference (>65%) for a particular compartment during the pretest, and two mice climbed out of the apparatus during the pretest or post-test. Sufficient numbers of mice of each sex were used to detect sex effects in behavioral tests (∼7) and in cotinine quantification experiments (∼5) when possible. Sex differences were always investigated using ANOVAs; data were collapsed across sex when a significant difference between sexes was not identified. Final sample sizes are included in the figure legends.

## Results

### Nicotine vapor delivers nicotine at behaviorally relevant concentrations

While it is known that systemic nicotine injections produce CPP/conditioned place aversion (CPA; Fudala et al., 1985; Jorenby et al., 1990; Torres et al., 2008), we wanted to assess the ability of nicotine vapor to produce these behaviors. We first validated a vapor delivery system that allowed the delivery of vapor to four chambers simultaneously (Fig. 1*A*). To ensure appreciable nicotine delivery using this method, we measured plasma cotinine concentrations in adolescent mice (P28 to P49) 30 min after nicotine vapor exposure at three e-liquid nicotine concentrations (2.5, 10, and 50 mg/ml). The resulting plasma cotinine concentrations were compared with those resulting from intraperitoneal nicotine injections, a traditional route of nicotine delivery in preclinical research (Fig. 1*B*). Nicotine injections consisted of a dose typically below the threshold needed to produce CPP (0.1 mg/kg nicotine), two doses previously shown to produce CPP (0.3 and 0.5 mg/kg nicotine), and a dose capable of producing CPA in mice (1.0 mg/kg nicotine; Fudala et al., 1985; Jorenby et al., 1990; Torres et al., 2008). Nicotine delivery via both routes of administration produced linearly increasing concentrations of plasma cotinine 30 min post-nicotine treatment in adolescent mice. Plasma cotinine concentrations achieved following vapor delivery were within a range known to be behaviorally relevant, as determined by cotinine concentrations measured in adolescent mice (Fig. 1*B*) at nicotine doses previously shown to affect behavior following injection (Fudala et al., 1985; Jorenby et al., 1990; Torres et al., 2008).

### Nicotine vapor produces CPP in adolescent mice

Next, we adapted our vapor delivery system for use in a novel vapor CPP protocol (Fig. 1*C*; see Materials and Methods). Briefly, during a pretest, we recorded baseline preferences for both compartments. Mice were then conditioned for 5 d, twice a day, with e-cigarette vapors. The time that mice spent in the treatment-paired compartment (CS+) before conditioning was compared with the time mice spent in the CS+ after conditioning. This widely used approach of comparing behavior before and after drug exposure allows us to detect changes in preference following conditioning (McKendrick and Graziane, 2020). A control group received vaporized vehicle containing equal parts VG and PG, industry-standard e-cigarette solvents, in both compartments.

Nicotine vapor produced significant CPP only at the lowest nicotine dose (2.5 mg/ml nicotine in e-liquid). At this dose, mice increased their time spent in the CS+ compartment relative to baseline (two-way repeated-measures ANOVA: *F*_(3,93)[nicotine concentration]_ = 0.127, *p* < 0.94; *F*_(1,93)[conditioning]_ = 16.25, *p* < 0.001; *F*_(3,93)[interaction]_ = 3.08, *p* < 0.05; Sidak’s multiple-comparison test: preconditioning vs postconditioning 2.5 mg/ml nicotine, *p* < 0.001). Conditioning with the vehicle vapor and with two higher concentrations of nicotine e-liquid (i.e., 10 and 50 mg/ml) did not affect time spent in the CS+ after conditioning (Fig. 1*D*).

### A strawberry additive is not rewarding

We next investigated the possible influence of the strawberry e-cigarette liquid additive on CPP. We first sought to identify the major chemical components found in the commercial strawberry additive used in these studies (Liquid Barn), which we expected would contain several volatile chemicals that together determine the “strawberry” odor profile. We sampled the volatiles from the strawberry additive using qualitative headspace GC/MS. This analysis uncovered nine primary constituents, including ethyl butyrate, 2-methyl-ethyl butyrate, benzyl acetate, propyl butyrate, 3-hexen-1-ol, 3-hexenyl acetate, linalool, menthyl acetate, and benzyl butyrate, in order of decreasing chromatographic peak responses (Extended Data Fig. 1-1). No nicotine was detected in the strawberry additive sample.

Before studying the possible effect of the strawberry additive on nicotine reward or aversion, we investigated its inherently rewarding or aversive properties by assaying the responses of mice to the additive-containing vapor with our CPP procedure. The vaporized strawberry additive did not produce CPP on its own regardless of concentration (2.5% or 10% of the e-liquid solution; two-way repeated-measures ANOVA: *F*_(2,58)[flavorant concentration]_ = 0.53, *p* = 0.59; *F*_(1,58)[conditioning]_ = 2.10, *p* = 0.15; *F*_(2,58)[interaction]_ = 0.56; *p* = 0.57; Fig. 1*E*).

### Strawberry additive does not alter nicotine CPP

We then assessed the effect of strawberry additive on nicotine preference. Preference scores were calculated by subtracting the time in the treatment-paired compartment (CS+) after conditioning from the time in the CS+ during the pretest. Preference scores were calculated so that the preference scores resulting from conditioning to nicotine only could be directly compared with those for vapor containing the same concentrations of nicotine plus strawberry additive (2.5% v/v) using a two-way ANOVA (Fig. 2). Strawberry additive did not significantly affect CPP for the nicotine-paired compartment (two-way ANOVA: *F*_(2,138)[nicotine concentration]_ = 4.80, *p* < 0.01; *F*_(1,138)[additive]_ = 3.70, *p* = 0.06; *F*_(2,138)[interaction]_ = 1.49, *p* = 0.23; Fig. 2).

### A strawberry additive in nicotine e-cigarette vapors leads to increased plasma cotinine concentrations

Despite its increased translational value, nicotine exposure via vapor is inherently more variable than via injections (e.g., because of individual differences in length and depth of inhalation), complicating attempts to standardize exposure. We therefore measured the plasma concentration of cotinine—the primary metabolite of nicotine—in mice undergoing CPP to obtain a more precise biological readout of nicotine delivery.

Surprisingly, despite the e-liquids containing the same concentration of nicotine, exposures to nicotine vapor containing a strawberry additive appeared to yield higher plasma cotinine levels at the highest nicotine concentrations (Fig. 3*A*). This phenomenon was not apparent at the lowest nicotine concentration (2.5 mg/ml), possibly reflecting the low precision of ELISA quantification at the cotinine levels produced by this nicotine concentration (e.g., 2.0–9.7 ng/ml). Thus, the 2.5 mg/ml nicotine concentration was excluded from our analyses.

There was a trend for increased plasma cotinine concentrations after exposure to vapor from 10 and 50 mg/ml nicotine e-liquid that also contained the strawberry additive, and a significant interaction between nicotine concentrations and the additive (Schreier–Ray–Hare, nicotine concentration * additive: *H*_[nicotine concentration]_ = 80.23, *p* < 0.0001; *H*_[additive]_ = 3.24, *p* = 0.07; *H*_[interaction]_ = 44.54, *p* < 0.0001; Fig. 3*A*). Mann–Whitney tests were used for further comparisons (Bonferroni-corrected α = 0.025). Strawberry additive only had a significant effect on plasma cotinine concentrations following the 10 mg/ml nicotine treatment (Mann–Whitney tests: 10 mg/ml nicotine vs 10 mg/ml nicotine plus strawberry, *p* < 0.01; 50 mg/ml nicotine vs 50 mg/ml nicotine plus strawberry, *p* = 0.11; Fig. 3*A*). The difference between the nicotine-only and nicotine plus additive groups at the 10 mg/ml nicotine concentration was “modest” (Cohen’s *d* = 0.58), whereas the effect at the 50 mg/ml nicotine concentration was “small” (Cohen’s *d* = 0.33; Cohen, 1988).

A difference in nicotine exposure levels could hypothetically alter the behavioral response to nicotine vapor with strawberry additive independent of the sensory and rewarding properties of the additive. In other words, it is possible that the negative trend of the additive on preference scores was related to increased nicotine intake (Fig. 2). To address this potential caveat, we sorted mice into cotinine categories based on plasma cotinine levels measured at the end of the vapor CPP experiment (see Materials and Methods, subsection Cotinine category determination). These cotinine categories allowed us to compare the behavior of mice in the additive-free and additive-containing nicotine groups that had similar levels of nicotine exposure. Interestingly, there was a negligible difference in preference scores between data categorized by concentration of nicotine in the e-liquid (Fig. 2) and data sorted by nicotine exposure levels (i.e., cotinine categories; data not shown). Given this result, we present the preference and aversion scores in Figure 2 based on the concentration of nicotine in the e-liquid, consistent with the rest of the article.

### Strawberry additive does not influence nicotine metabolism

We next considered the potential mechanisms underlying the effect of the strawberry additive in the e-liquid on levels of nicotine exposure (i.e., cotinine; Fig. 3*A*). One possibility is that the additive interferes with normal nicotine metabolism and, consequently, plasma cotinine levels. To test this hypothesis, we injected adolescent mice with either 1.0 mg/kg, i.p., nicotine in a 100% PBS vehicle or 1.0 mg/kg nicotine in a PBS solution containing 2.5% strawberry additive. There was a significant effect of sex on cotinine concentrations following nicotine injection that was not eliminated by normalizing by weight, thus sex was included as an interaction term. Inclusion of the strawberry additive did not affect plasma cotinine concentrations 30 min postinjection (two-way ANOVA sex * additive: *F*_(1,21)[sex]_ = 25.50, *p* < 0.0001; *F*_(1,21)[additive]_ = 0.89, *p* = 0.36; *F*_(1,21)[interaction]_ = 0.01, *p* = 0.92; Fig. 3*B*). These results suggest that the strawberry additive did not influence nicotine metabolism.

### Addition of a strawberry e-cigarette additive promotes the inhalation of nicotine vapor

We next evaluated the possibility that mice were interacting with strawberry-flavored vapors differently; perhaps they inhaled more often when the e-cigarette vapor included the strawberry additive or reduced the inhalation of unflavored nicotine because of its properties as an irritant (Oliveira-Maia et al., 2009). To test these hypotheses, we used a plethysmograph to measure respiration in mice while they sampled nicotine vapors (Fig. 4*A*,*B*). Notably, in this design, e-cigarette liquids were not heated in an effort to more directly investigate olfactory sources of possible reward or aversion, since heating results in a more multisensory stimulus because of the sounds of the heating, visual cues of a plume, and temperature changes. Mice were first habituated to the residual sensory cues of stimuli being delivered into the plethysmograph by repeatedly delivering 50:50 VG/PG vehicle (Fig. 4*C*) into the plethysmograph over 2 d. On the third day, we delivered pseudorandom trials of e-cigarette odors while simultaneously measuring sniffing (Fig. 5).

Vapors from all e-cigarette liquids elicited fast exploratory sniffing on their first presentation (Fig. 5*A*,*B*). Importantly, this indicates that the mice perceived all vapors, even nicotine when presented alone. As expected (Sundberg et al., 1982; Wesson et al., 2008), over repeated trials of odor delivery, mice habituated their sniffing response to most but not all vapors. This sustained sniffing response is meaningful in the present context since mice sniff odors they perceive as attractive more than odors they do not (Baum and Keverne, 2002; Jagetia et al., 2018).

We calculated the amount of time the mice spent in high-frequency investigatory sniffing (>6 Hz; Wesson et al., 2008) on the final presentation of each vapor and found that many of the mice studied engaged in more sniffing during the final presentations of vapors containing strawberry additive alone or nicotine when it contains strawberry additive (Fig. 5*B*). We detected a main effect of strawberry additive on investigatory sniffing toward nicotine vapor (two-way ANOVA, nicotine * additive: *F*_(1,24)[additive]_ = 8.36, *p* < 0.01; *F*_(1,24)[nicotine]_ = 0.50, *p* = 0.49; *F*_(1,24)[interaction]_ = 2.97, *p* = 0.10), with subsequent *post hoc* tests revealing that this effect was specific to the 50 mg/ml nicotine e-liquid (Sidak’s multiple-comparisons test, *p* < 0.001; Fig. 5*C*). There were no differences in the amplitude of sniffing between the different e-cigarette vapors (*p* > 0.05; data not shown). These results indicate that mice actively inhale strawberry containing e-cigarette odor more than they do e-cigarette odor with nicotine alone.

## Discussion

We used a vapor delivery system to investigate how a commercial e-cigarette additive (i.e., strawberry flavor) might impact nicotine-associated behaviors. Our observations suggest that a strawberry additive may increase exposure to nicotine by increasing the interaction with nicotine vapor rather than altering reward. Our results support the hypothesis that additives with significant chemosensory attributes, specifically those fruit like in their composition, promote nicotine intake.

### Adolescent mice express conditioned reward in response to nicotine vapor

We found that nicotine vapor leads to CPP in adolescent mice at a low dose of nicotine (2.5 mg/ml nicotine in e-liquid; Fig. 1*D*). Interestingly, our results suggest that, when nicotine is delivered via inhalation, reward may occur at lower levels of exposure than previously thought. We only observed an increase in time spent in the CS+ following conditioning with a nicotine treatment that yielded plasma cotinine levels (∼8 ng/ml) that were most likely lower than those observed when measuring CPP in response to nicotine injection (0.3 and 0.5 mg/kg; Fudala et al., 1985; Jorenby et al., 1990; Torres et al., 2008). These previously published studies did not measure plasma cotinine concentrations. However, in our experiments, when we measured cotinine levels following intraperitoneal injections of 0.3 and 0.5 mg/kg we observed plasma cotinine concentrations of ∼46 and ∼76 ng/ml, respectively (Fig. 1*B*). The level of CPP observed here is modest, but is consistent with that reported in CPP experiments using systemic injections (Brielmaier et al., 2008; Brunzell et al., 2009; Sershen et al., 2010). The detection of CPP at the lowest nicotine vapor concentration could be because of the increased rate of nicotine delivery to the brain when inhaled rather than injected, which can affect subjective reward (Samaha and Robinson, 2005; Jensen et al., 2020). However, at least one other study has also demonstrated CPP in adolescents at a seemingly subthreshold dose (i.e., 0.1 mg/kg nicotine, s.c.; Ahsan et al., 2014).

An important consideration is that mice and humans interact differently with e-cigarette vapors. In humans, e-cigarette use is highly multisensory: users inhale vapors orally (resulting in the perception of taste, temperature, and retronasal olfaction) as well as nasally, which together provide important information on flavor. In contrast, mice are preferentially nasal breathers, so that while some exposure occurs orally, the majority occurs nasally (Agrawal et al., 2008). Trigeminal fibers are also present in the mouse nose, where they detect pungent stimuli (e.g., high concentrations of nicotine vapor), together with the olfactory receptors that detect volatile compounds, including nicotine and the chemicals found in vapor additives (Extended Data Fig. 1-1). Thus, although our data do not perfectly reproduce the human experience, they nevertheless provide relevant information on how fruity additives might alter the perception of nicotine vapor in humans.

There has been a growing interest in the field of nicotine research to deliver nicotine as a vapor in preclinical models to study the rewarding and reinforcing properties of this drug that humans typically inhale. However, to our knowledge, this is the first study to use e-cigarette vapor to condition animals to nicotine in a pavlovian CPP paradigm. The pharmacokinetics of nicotine delivered by inhalation differs significantly from that of nicotine delivered via injection. By using nicotine vapor, our goal was to improve the translatability of our model and possibly improve our ability to detect nicotine reward. It was necessary to condition mice in air-tight compartments to ensure equal e-cigarette vapor distribution and protect researchers from harmful vapors. However, an open chamber was required for the pretests/post-tests to record the movements of animals from above. Although the testing and training apparatuses were technically different objects, precautions were taken to ensure that the conditioning and testing chambers were indistinguishable from one another to mice. First, all chambers were made from the same clear Plexiglas material, and the airtight conditioning chambers were the exact dimensions as one-half of the testing chamber. Importantly, the visual and tactile cues used in the CPP experiments were removable. This allowed the same visual and tactile cues to be used in the conditioning and testing chambers. These cues are the most distinctive features present during conditioning and are most likely to have become associated with the physiological effects of drug treatment. It is still possible that mice were able to perceive subtle differences between the testing and conditioning chambers. Still, we expect that by using the same cues, we were able to mostly avoid possible confounds.

### A strawberry additive does not alter the expression of nicotine vapor reward

We did not find evidence that the strawberry additive altered adolescent nicotine reward; however, methodological factors may have affected this. For example, we chose to measure the effect of a relatively low concentration of strawberry additive (2.5% v/v) based on the sensitive olfactory system of the mouse and on our finding no significant effect on preference scores at 10% (Fig. 1*F*,*G*). Additives in the e-liquids used by human vapers, however, are often present at much higher concentrations—up to twice that of the nicotine present (Omaiye et al., 2019). It is possible that, at higher additive concentrations, more volatiles would be transferred into the e-cigarette vapor and absorbed, where they might have pharmacological effects capable of modifying nicotine reward (Avelar et al., 2019).

Consumer e-cigarette products emit a vast number of volatiles. Indeed, a comprehensive study of 277 commercial e-liquids identified 155 unique sensory volatiles, with an average e-liquid containing ∼25 chemicals (Omaiye et al., 2019). Our finding that a single commercial e-liquid, dominated in its volatile composition by only nine chemicals, does not alter nicotine reward or aversion should not be generalized. It is possible that other individual flavor components, such as farnesol, or even the same chemicals found in strawberry—at different concentrations or in combination with others—could alter the response to nicotine and affect nicotine CPP. Indeed, farnesol, an e-cigarette additive that mimics “green apple” flavor, can enhance nicotine reward in male mice (Avelar et al., 2019). The difference between our report and the study by Avelar et al. (2019) might also reflect differences in pharmacology between farnesol and the volatiles present in the strawberry additive studied here or, alternatively, could result from the different methods used. Avelar et al. (2019) injected nicotine and farnesol rather than delivering them as vapor, potentially leading to significantly higher farnesol absorption levels than those reached via inhalation. In addition, pyrolysis during vaping might generate compounds with different pharmacological properties than those of the original additive.

### Strawberry additive increases nicotine exposure in adolescent mice

Mice exposed to strawberry additive-containing nicotine vapor had higher plasma cotinine concentrations than those exposed to nicotine alone. We first considered the possibility that the strawberry additive could affect nicotine metabolism and result in increased plasma levels of cotinine, the primary metabolite of nicotine. In fact, linalool—a common volatile compound in e-cigarette flavors, which we detected as a constituent in the Liquid Barn additive (Extended Data Fig. 1-1)—affects cytochrome P450 activity in the rat liver, albeit only at very high doses (Nosková et al., 2016). However, when we injected nicotine with or without the presence of the strawberry additive, we did not observe a meaningful effect on nicotine metabolism and plasma cotinine levels, suggesting that altered metabolism is not driving a difference in plasma cotinine levels.

Additives may also alter nicotine absorption through changes in pH of e-liquid solutions and vapors (St. Helen et al., 2017, 2018; Patten and De Biasi, 2020), as has been previously reported in humans (St. Helen et al., 2017, 2018). Nicotine absorption is facilitated by more acidic vapors, which allow a larger proportion of the nicotine to reach the lung where it can be buffered, absorbed, and rapidly delivered to the brain (Patten and De Biasi, 2020). Although we did not explicitly test for pH effects, we observed a trend for a slightly lower pH in nicotine e-liquids containing the strawberry flavorant (Extended Data Fig. 1-2), which would increase the percentage of protonated nicotine at every related experimental condition. Thus, it is possible that more acidic strawberry e-liquids/vapors lead to an increase in nicotine absorption, contributing to higher plasma cotinine concentrations (Fig. 3*A*).

Although altered chemical properties of e-cigarette vapors may have contributed to higher nicotine exposure, our study suggests that chemosensory-rich additives found in e-cigarette liquids alter the way the mice interact with e-cigarette vapors. Additives appear to influence vaping topography in humans (Audrain-McGovern et al., 2016; St. Helen et al., 2018). For example, St. Helen et al. (2018) measured a longer average puff duration when participants vaped a strawberry e-liquid compared with a tobacco e-liquid. This phenomenon is recapitulated by our results demonstrating increased time spent sniffing vapors containing the strawberry additive, which may contribute to the increased nicotine intake we observed. By lowering the pH, the strawberry flavor additive increases the percentage of nicotine salt/protonated nicotine, which is smoother, less harsh, and decreases throat hit. This phenomenon would make vaping higher-concentration nicotine liquids less unpleasant (Gholap et al., 2020). The observed effect size in our study ranges from relatively small to modest; nevertheless, the inclusion of the odorant increased the level of plasma cotinine to between 114% (10 mg/ml) and 18% (50 mg/ml). Given the popularity of flavored e-cigarette liquids, even a seemingly small difference may have a profound impact at a population level, and a small increase in nicotine exposure may be sufficient to increase the susceptibility to or rate of developing dependence (DiFranza et al., 2007; MacPherson et al., 2008), including within days or weeks of inhaling their first cigarette (DiFranza et al., 2007). Detecting early symptoms of nicotine dependence is important because it may help identify individuals who are on trajectories toward heavier tobacco use (Lessov-Schlaggar et al., 2008).

Our preclinical results add to the argument that additives with significant chemosensory attributes, specifically those fruit like in their composition, influence the intake of e-cigarette vapors in manners that promote nicotine intake. Because mice are preferential nasal breathers (Agrawal et al., 2008), we predict that the olfactory components of the additive greatly mediate this effect. However, additional work is needed to resolve the sensory influences of this effect.

### Conclusion

Our results indicate that a strawberry fruit additive, one of many in a category of highly used “e-cigarette flavors,” significantly increases nicotine exposure but not reward. This finding is particularly important for understanding e-cigarette effects on adolescents who are vulnerable to nicotine uptake and whose brains are sensitive to long-term disruptions mediated by nicotine exposure at this age (DiFranza et al., 2007; Substance Abuse and Mental Health Services Administration, 2011; Goriounova and Mansvelder, 2012; Yuan et al., 2015). Our data also highlight an underappreciated role of e-cigarette flavor additives: their ability to alter one’s response to nicotine via sensory contributions, possibly leading to differences in nicotine exposure. Further research using models of nicotine self-administration with a fruit-flavored or sweet-flavored vapor may help predict the ultimate impact of increased nicotine intake under these conditions.

## Synthesis

Reviewing Editor: Julie Bakker, University of Liege Decisions are customarily a result of the Reviewing Editor and the peer reviewers coming together and discussing their recommendations until a consensus is reached. When revisions are invited, a fact-based synthesis statement explaining their decision and outlining what is needed to prepare a revision will be listed below. The following reviewer(s) agreed to reveal their identity: NONE.

synthesis: the paper has been reviewed by 1 reviewer, great difficulty unfortunately for getting a second reviewer to agree. However, I agree with the comments of the first reviewer, I have therefore decided to go with this review in order not to lose any more time. Please follow closely his/her suggestions when revising your paper

Reviewer 1:

A major strength of this work is this is the first reported CPP paradigm paired with vapor inhalation of nicotine within the same chamber (at least this reviewer does not know of the existence of any other reports). One downside of using commercial e-liquids or commercial concentrates is the variability but the authors remove this weakness by assessing the constituents of their e-liquid. Accordingly, this is a strength as many investigators are unable to report the precise chemical constituents of their e-liquids. The concept that flavors may not impact reward but facilitate increased inhalation of nicotine-containing vapor via inhalation frequency is highly novel and will be very informative to the field. Despite this, there are some serious weaknesses in the CPP paradigm. Specifically in the description of how the assay is being completed and the statistics used to determine nicotine’s significant effect. Overall, this detracts from the overall impact of this work.

Major Comments:

Some technical details need to be added

1. What was the resistance of the coils used in the SMOK Baby Beast tanks?

2. What temperature setting was used for the LJARI controllers?

One technical detail may have been overlooked. The authors completed their validation of plasma cotinine levels using a single tank that delivers to 4 separate chambers. Following this, they then used a single tank to deliver the exact same puff to a single chamber for CPP. It is possible in the smaller volume may produce a higher pharmacokinetic profile of nicotine and flavorants in the CPP paradigm compared to the validation paradigm. The authors should address this.

Additional technical details should be provided for the CPP-adapted vapor exposure system. In the diagram of Figure 1, it appears that the delivery into the CPP conditioning chambers is not into an airtight system. However, the methods do state that the airtight chambers are adapted for the paradigm. This then infers that conditioning was not done in an actual CPP chamber but in an air-tight vapor inhalation chamber that is distinctly different from the chamber used for exploration during the pre-test and post-test. Traditionally, CPP exploration (Pre/post-test) and conditioning occurs all within the same chamber. The establishment of a preference in one chamber and then conditioning in a separate chamber adds a major confound to the paradigm that is difficult to address. The authors need to be very clear in the precise setup that they are using to examine vapor-inhalation CPP. One thing that confuses this matter is the diagram provided in Figure 1C that makes one believe that an existing CPP chamber is just modified to facilitate vapor-inhalation but that appears to not be how this is done in reality. The schematic in Figure 1 needs to be adapted to more-closely resemble the experimental setup. Alternatively, the authors can provide an actual photo of the setup to clearly provide what this actually looks like. Also, if the authors are completing conditioning and exploration in separate chambers, they need to address the limitations of doing this in the discussion section.

The authors can’t really conclude that nicotine vapor produced significant CPP as their assumption is based upon using a one-sample t-test. For CPP as presented here (control vs. multiple drug concentrations) it is typical to use a one-way ANOVA with an appropriate post hoc comparison and not a one-sample t-test. The authors actually did this comparison (ANOVA with Tukey) to compare the 2.5 and 50 mg/mL conditions so the same appropriate statistics need to be employed to determine if nicotine has an effect when compared to PGVG.

Minor Comments:

While pH was not adjusted, the authors may want to report what the pH values of their e-liquids are. Flavors can change the pH drastically and the pH will determine the proportion of protonated/unprotonated nicotine ions.

In figure 2’s legend, the concentration of the additive should be provided.
