# Supplementary figures and images for: Strawberry Additive Increases Nicotine Vapor Sampling and Systemic Exposure But Does Not Enhance Pavlovian-Based Nicotine Reward in Mice
Source: eNeuro. 2023 Jun 12;10(6):ENEURO.0390-22.2023. doi: 10.1523/ENEURO.0390-22.2023 (PMC10275399; doi:10.1523/ENEURO.0390-22.2023)

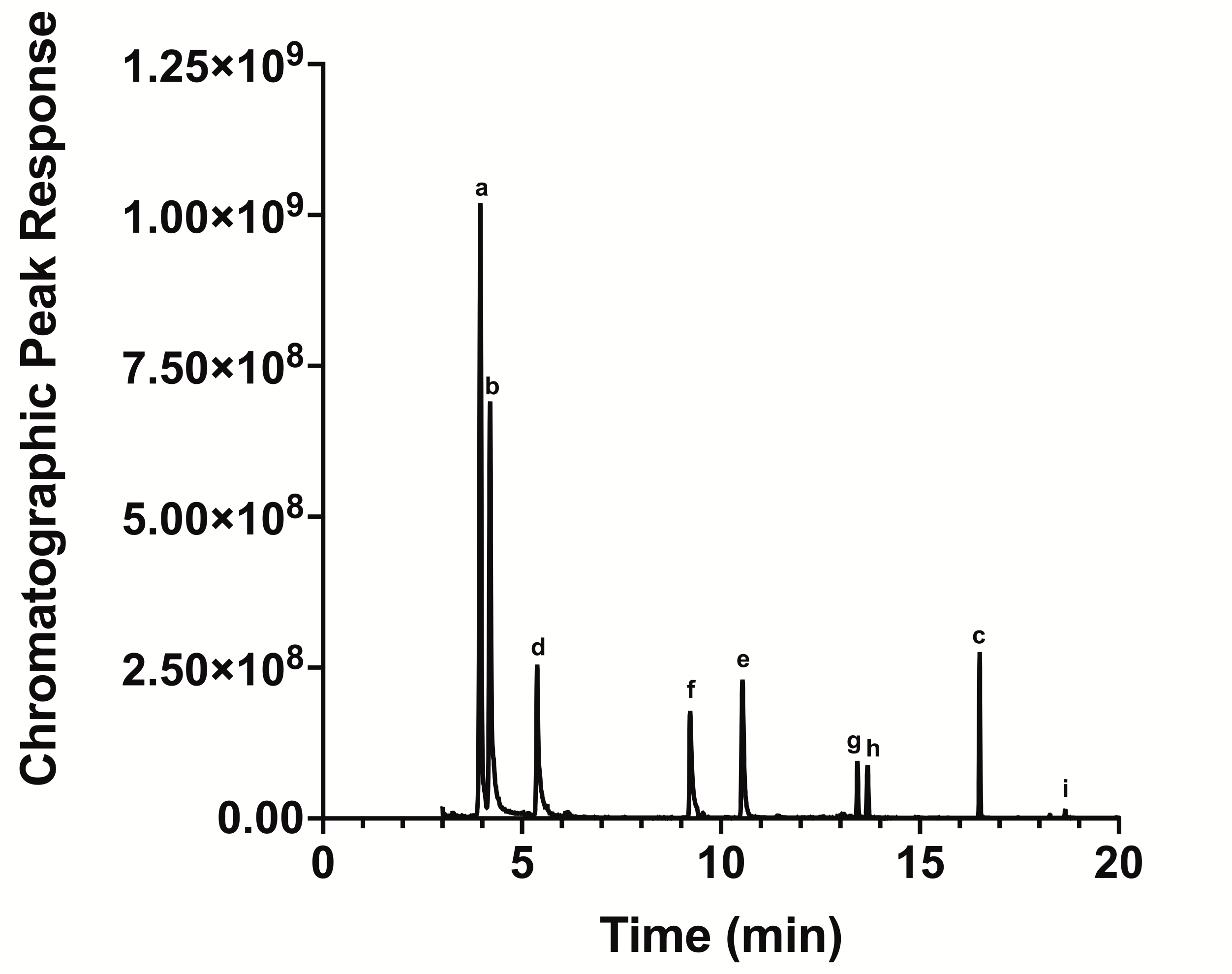

Supplement: Figure 1-1 — GC/MS detects chemical volatiles in strawberry e-liquid. The chromatogram from GC/MS analysis of 0.1% “Strawberry Flavor Concentrate” (Liquid Barn) in water shows the following chemicals dominate in the commercial e-liquid headspace in order from highest to lowest chromatographic peak responses: (a) ethyl butyrate, (b) 2-methyl-ethyl butyrate, (c) benzyl acetate, (d) propyl butyrate, (e) 3-hexen-1-ol, (f) 3-hexenyl acetate, (g) linalool, (h) menthyl acetate, and (i) benzyl butyrate. Download Figure 1-1, TIF file. [file enu-eN-NWR-0390-22-s01.tif]
