# Supplementary material for: Strawberry Additive Increases Nicotine Vapor Sampling and Systemic Exposure But Does Not Enhance Pavlovian-Based Nicotine Reward in Mice
Source: eNeuro. 2023 Jun 12;10(6):ENEURO.0390-22.2023. doi: 10.1523/ENEURO.0390-22.2023 (PMC10275399; doi:10.1523/ENEURO.0390-22.2023)
Supplement: Figure 1-2 — pH of the e-liquids utilized in this study estimated by assuming [H+] in aqueous solution. Briefly, e-liquids were made in a 50:50 VG/PG blend with nicotine dissolved in the VG portion and additive dissolved in the PG portion. To reduce the viscosity of the e-liquid and in order to mimic the protocol used in the study by St. Helen et al. (2017), a 1:10 e-liquid-to-Milli-Q water dilution of each e-liquid was prepared. pH test strips were then used to estimate the pH. Download Figure 1-2, DOC file. [file enu-eN-NWR-0390-22-s03.doc]

| **E-liquid** | **estimated pH** |
| --- | --- |
| Vehicle (50/50 VG/PG) | 4.5 |
| 2.5% strawberry additive | 4 |
| 2.5 mg/ml nicotine | 7.4 |
| 2.5 mg/ml nicotine + 2.5% strawberry additive | 7.1 |
| 10 mg/ml nicotine | 8.3 |
| 10 mg/ml nicotine + 2.5% strawberry additive | 8.1 |
| 50 mg/ml nicotine | 9.0 |
| 50 mg/ml nicotine + 2.5% strawberry additive | 8.7 |

Figure 1-2. pH of the e-liquids utilized in this study estimated by assuming [H^+^] in aqueous solution. Briefly, e-liquids were made in a 50/50 Vegetable (VG) Glycerin/Propylene Glycol (PG) blend with nicotine dissolved in the VG portion and additive dissolved in the PG portion. To reduce the viscosity of the e-liquid and in order to mimic the protocol used in St. Helen, et al., 2017 (DOI: [10.1016/j.drugalcdep.2017.05.042](https://doi.org/10.1016/j.drugalcdep.2017.05.042)), a 1:10 e-liquid to milliQ water dilution of each e-liquid was prepared. pH test strips were then used to estimate the pH.
